# Supplementary figures and images for: Expansion of the geranylgeranyl pyrophosphate synthase gene family underlies the evolution of terpenoid biosynthesis in termites
Source: PLoS Biol. 2026 Apr 27;24(4):e3003648. doi: 10.1371/journal.pbio.3003648 (PMC13143122; doi:10.1371/journal.pbio.3003648)

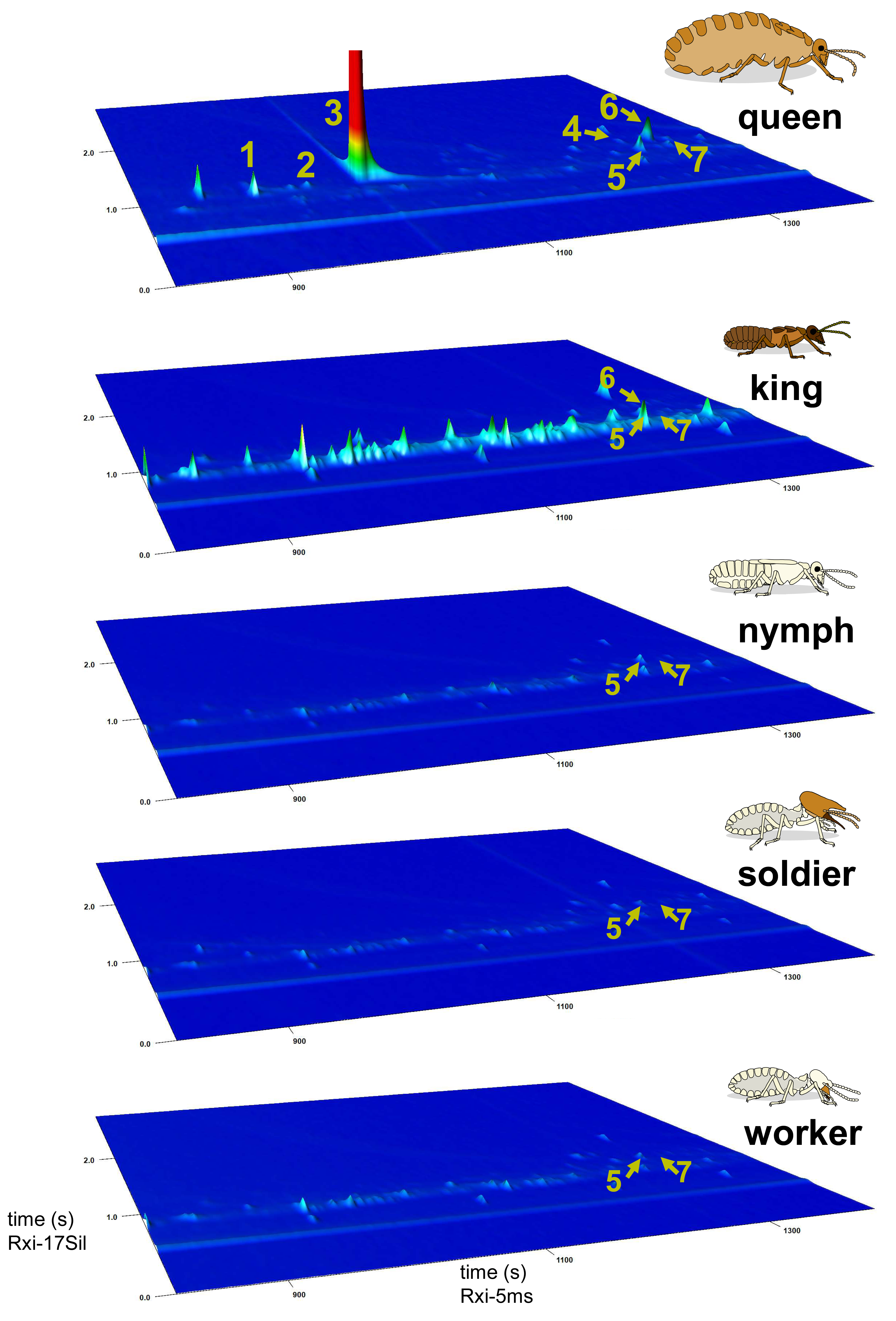

Supplement: S1 Fig — The chromatograms show the retention window where terpenoids were detected. 1, β-farnesene; 2, α-farnesene; 3, (6E)-nerolidol; 4, unidentified diterpene; 5, β-springene; 6, (E,E,E)-neocembrene, 7, unidentified diterpene. The chromatogram visualizes the responses to diagnostic ions for mono-, sesqui-, and diterpenes (m/z = 68 + 69 + 93 + 204 + 272). Mass spectra of the identified terpenes are shown below in S2 Fig. Complete chromatographic data are available from OSF (https://osf.io/rkdy9). (TIF) [file pbio.3003648.s001.tif]

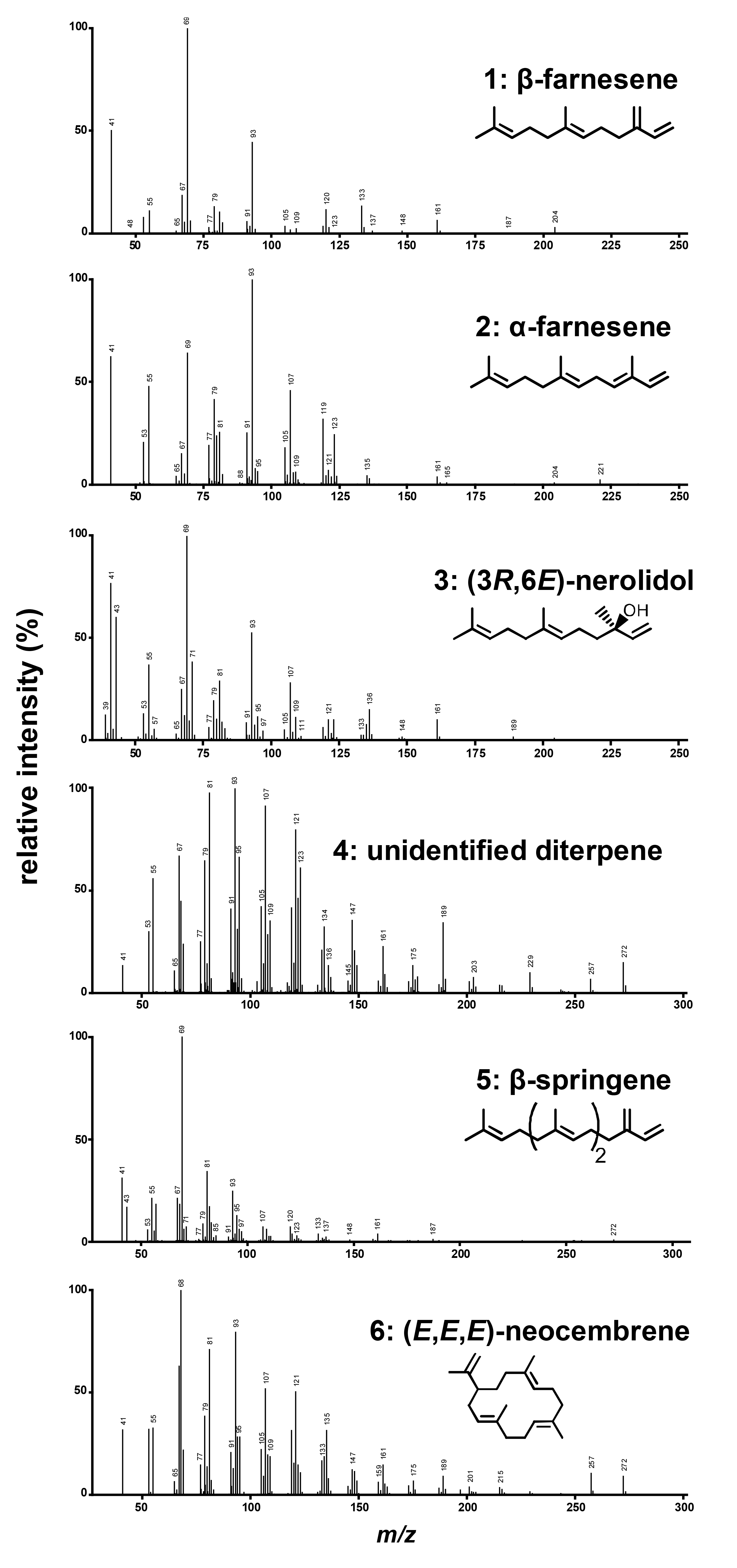

Supplement: S2 Fig — Compound numbering refers to that in Figs 1 and S1. For compound 7 (unidentified diterpene), we could not obtain a reliable mass spectrum. (TIF) [file pbio.3003648.s002.tif]

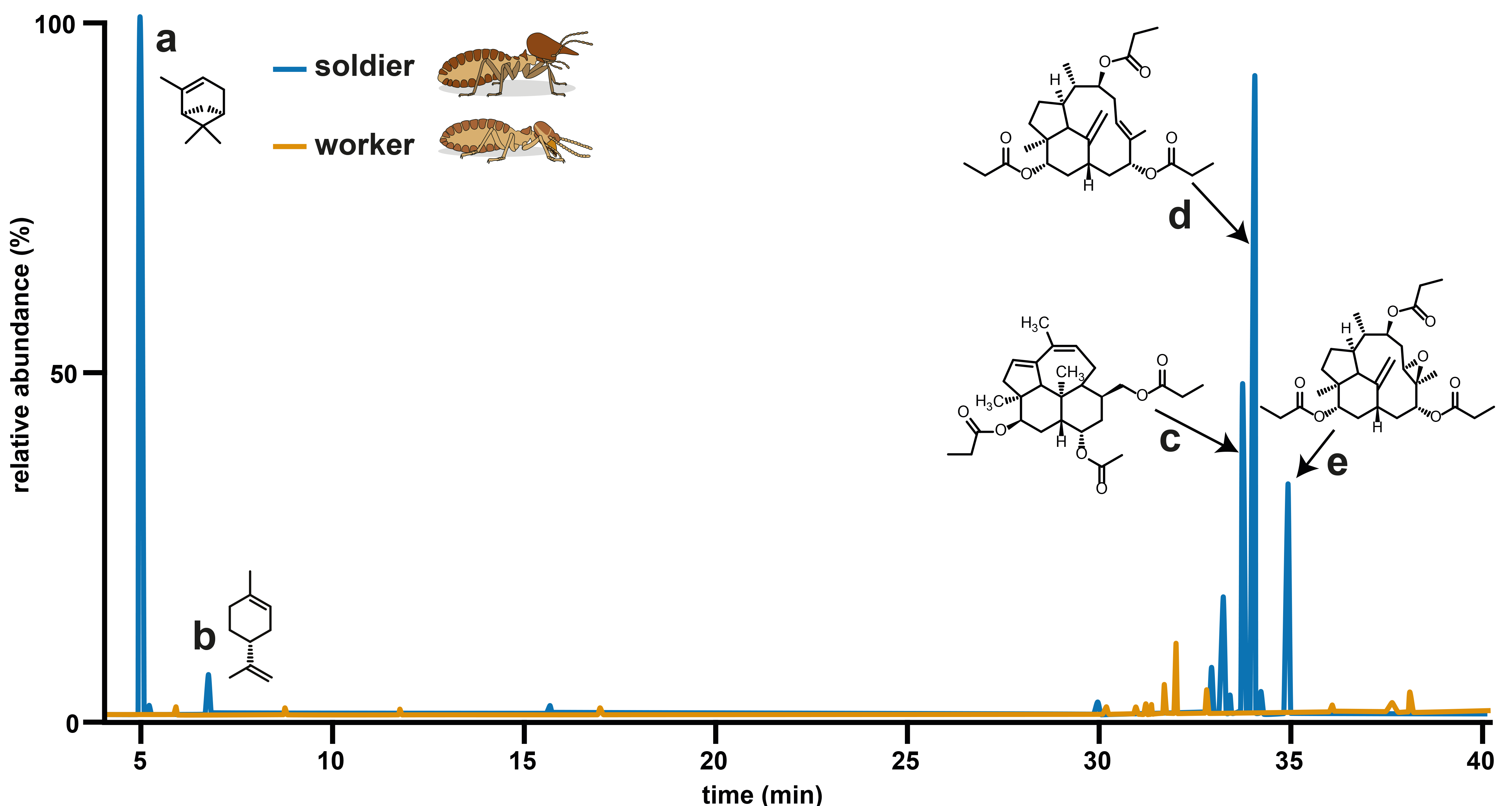

Supplement: S3 Fig — The chromatogram visualizes the responses to diagnostic ions for mono-, sesqui-, and diterpenes (m/z = 68 + 69 + 93 + 204 + 272). Mass spectra and NMR spectra of the identified terpenes are shown below in S4–S8 Figs. Complete chromatographic data are available from OSF (https://osf.io/rkdy9). (TIF) [file pbio.3003648.s003.tif]

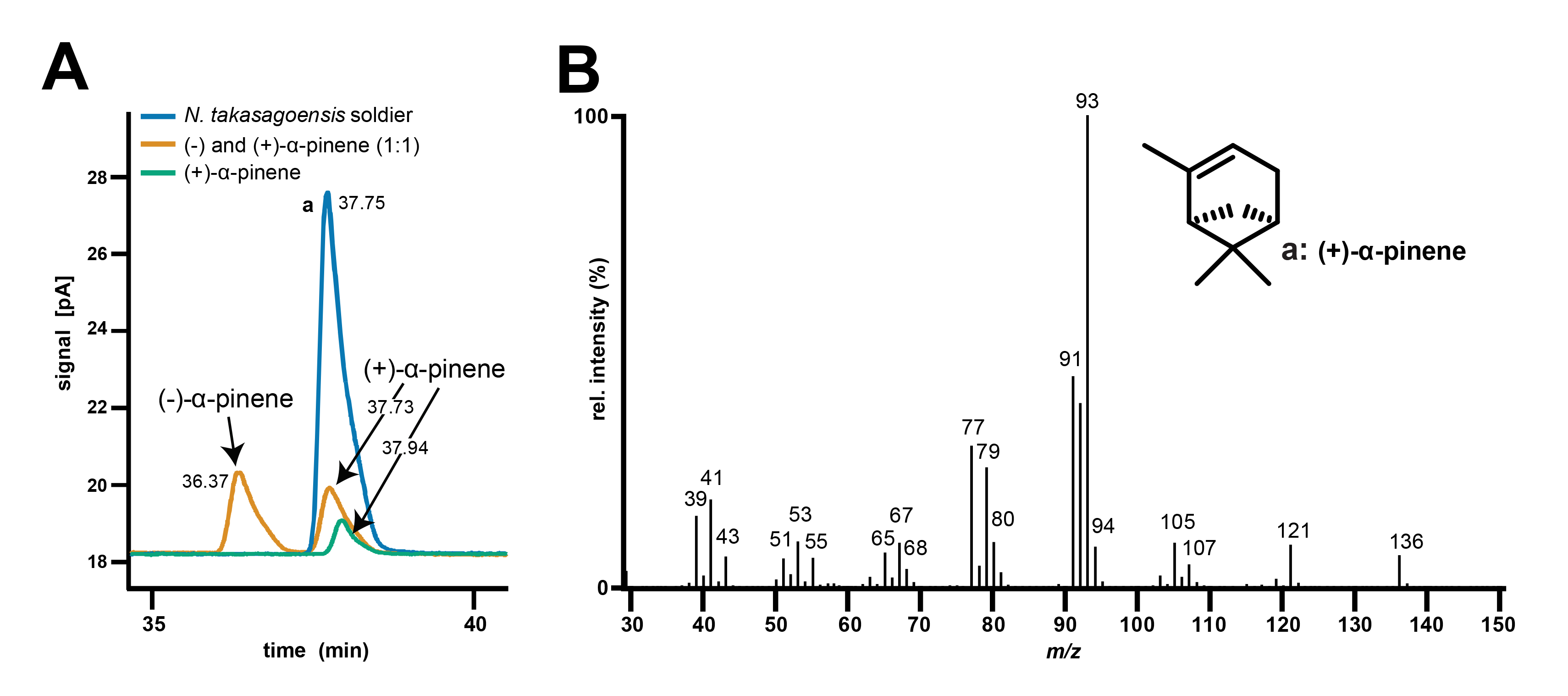

Supplement: S4 Fig — A. Chiral chromatogram comparing the retention of compound a with standards of (−) and (+)-α-pinene. GC-FID was equipped with cyclodextrin chiral column (HP CHIRAL β, 30 m, 0.25 mm, 0.25 µm, 19091G-B233E). One µL of sample was injected in splitless mode following this temperature program: 40 °C held for 20 min, ramped 1 °C/min to 100 °C, ramp at 30 °C/min to 240 °C, held for 20 min. B. Mass spectrum of compound a. Complete chromatographic data are available from OSF (https://osf.io/rkdy9). (TIF) [file pbio.3003648.s004.tif]

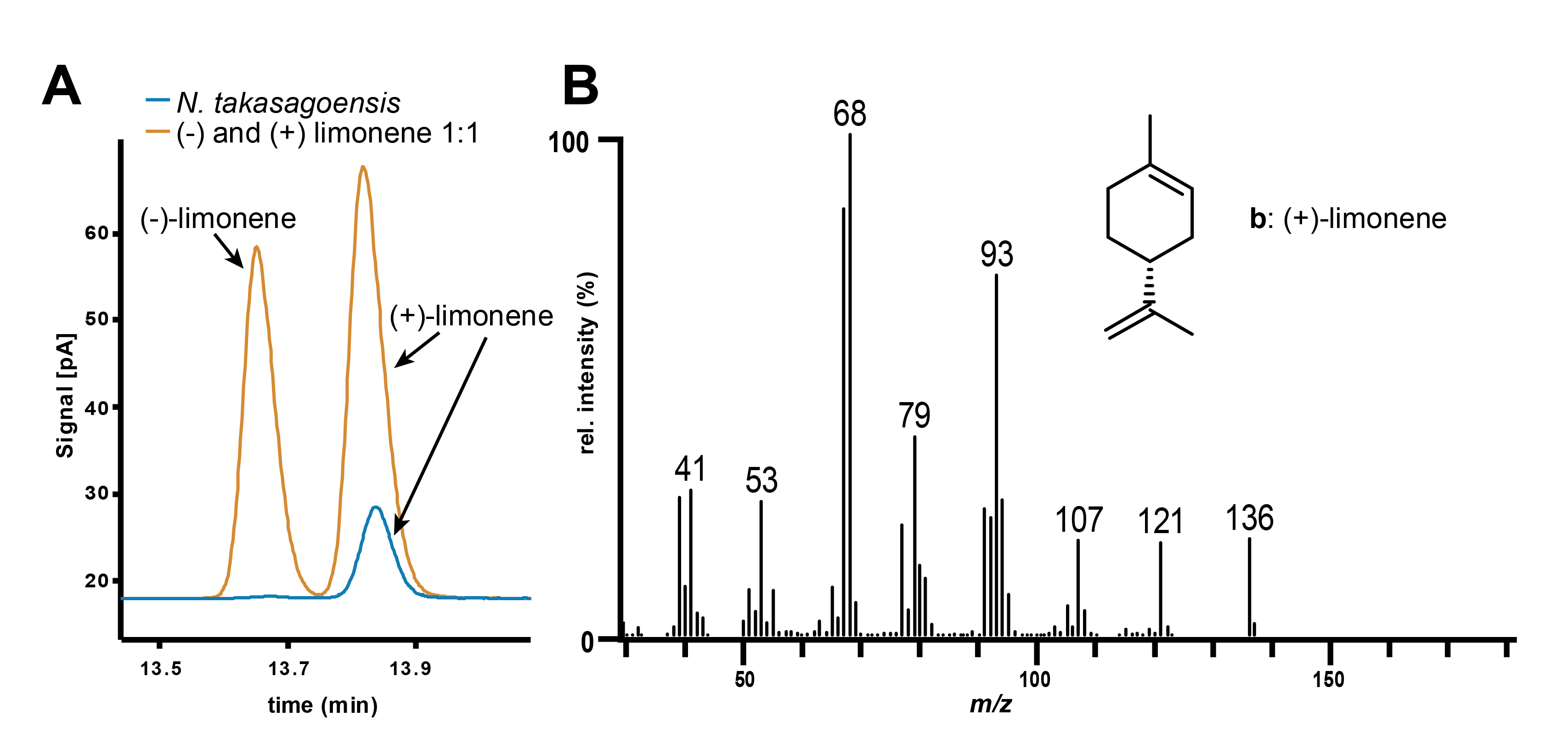

Supplement: S5 Fig — A. Chiral chromatogram comparing the retention of compound b with standards of (−) and (+)-limonene. GC-FID was equipped with cyclodextrin chiral column (HP CHIRAL β, 30 m, 0.25 mm, 0.25 µm, 19091G-B233E). One µL of sample was injected in splitless mode following this temperature program: 40 °C held for 1 min, ramped 3 °C/min to 130 °C, ramp at 30 °C/min to 240 °C, held for 10 min. B. Mass spectrum of compound b. Complete chromatographic data are available from OSF (https://osf.io/rkdy9). (TIF) [file pbio.3003648.s005.tif]

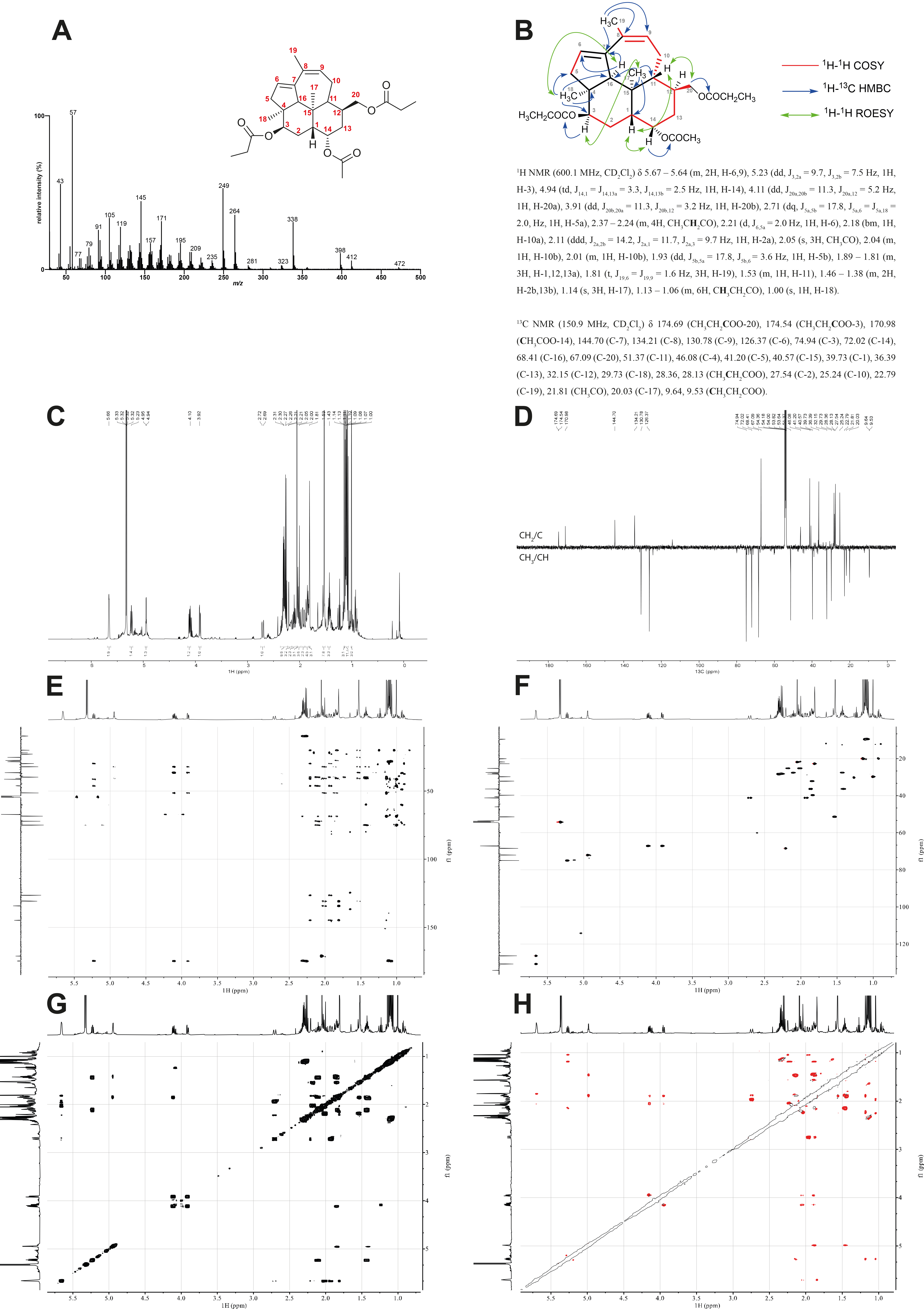

Supplement: S6 Fig — A. Mass spectrum. B. Key 2D NMR correlations and assigned 1H and 13C NMR spectra. C. 1H NMR spectra. D. APT NMR spectrum. E. HMBC NMR spectra. F. HSQC NMR spectrum. G. COSY NMR spectrum. H. ROESY NMR spectrum. Complete NMR data are available from OSF (https://osf.io/rkdy9). (TIF) [file pbio.3003648.s006.tif]

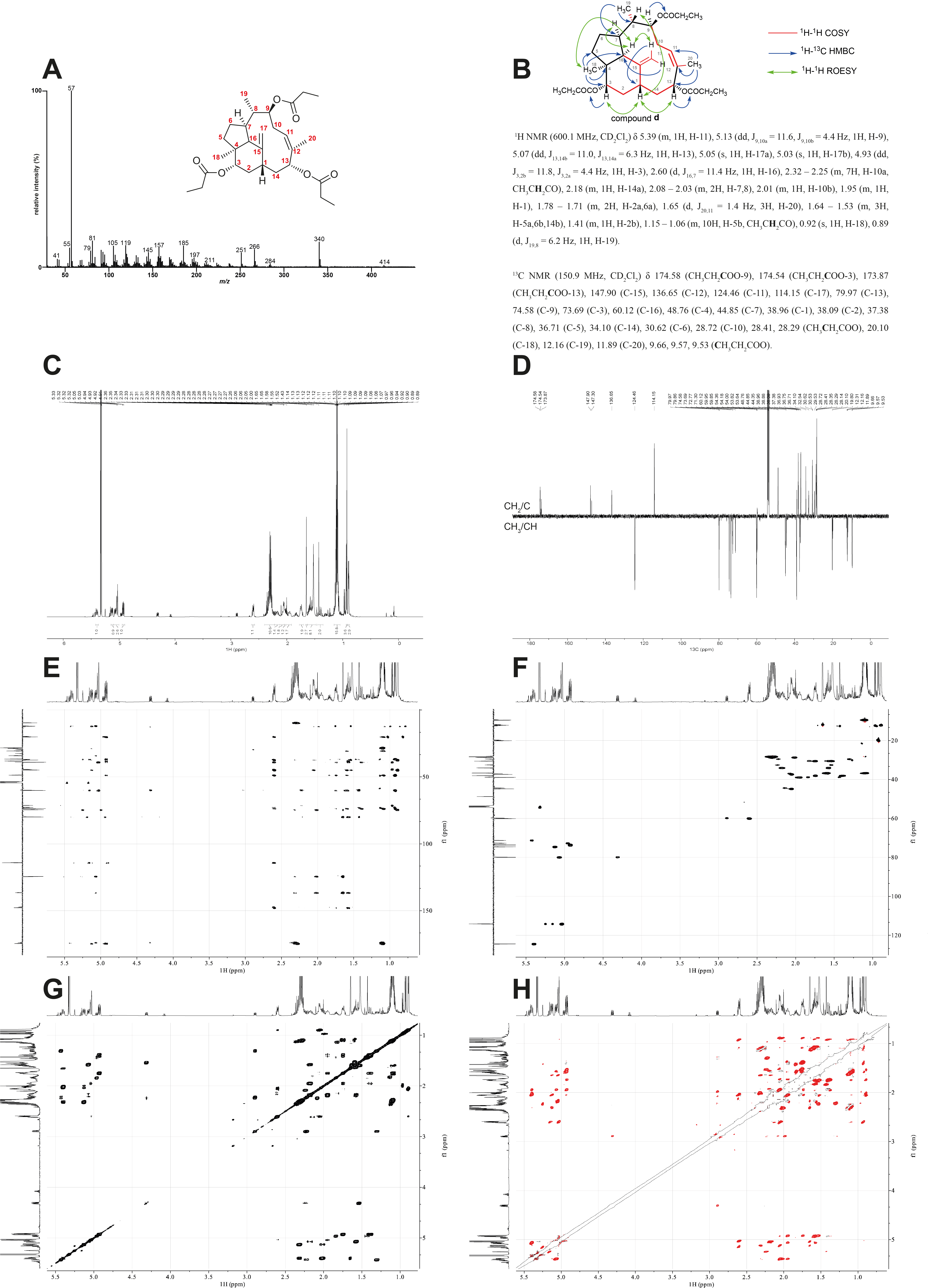

Supplement: S7 Fig — A. Mass spectrum. B. Key 2D NMR correlations and assigned 1H and 13C NMR spectra. C. 1H NMR spectra. D. APT NMR spectra. E. HMBC spectra. F. HSQC NMR spectra. G. COSY spectra. H. ROESY NMR spectra. Complete NMR data are available from OSF (https://osf.io/rkdy9). (TIF) [file pbio.3003648.s007.tif]

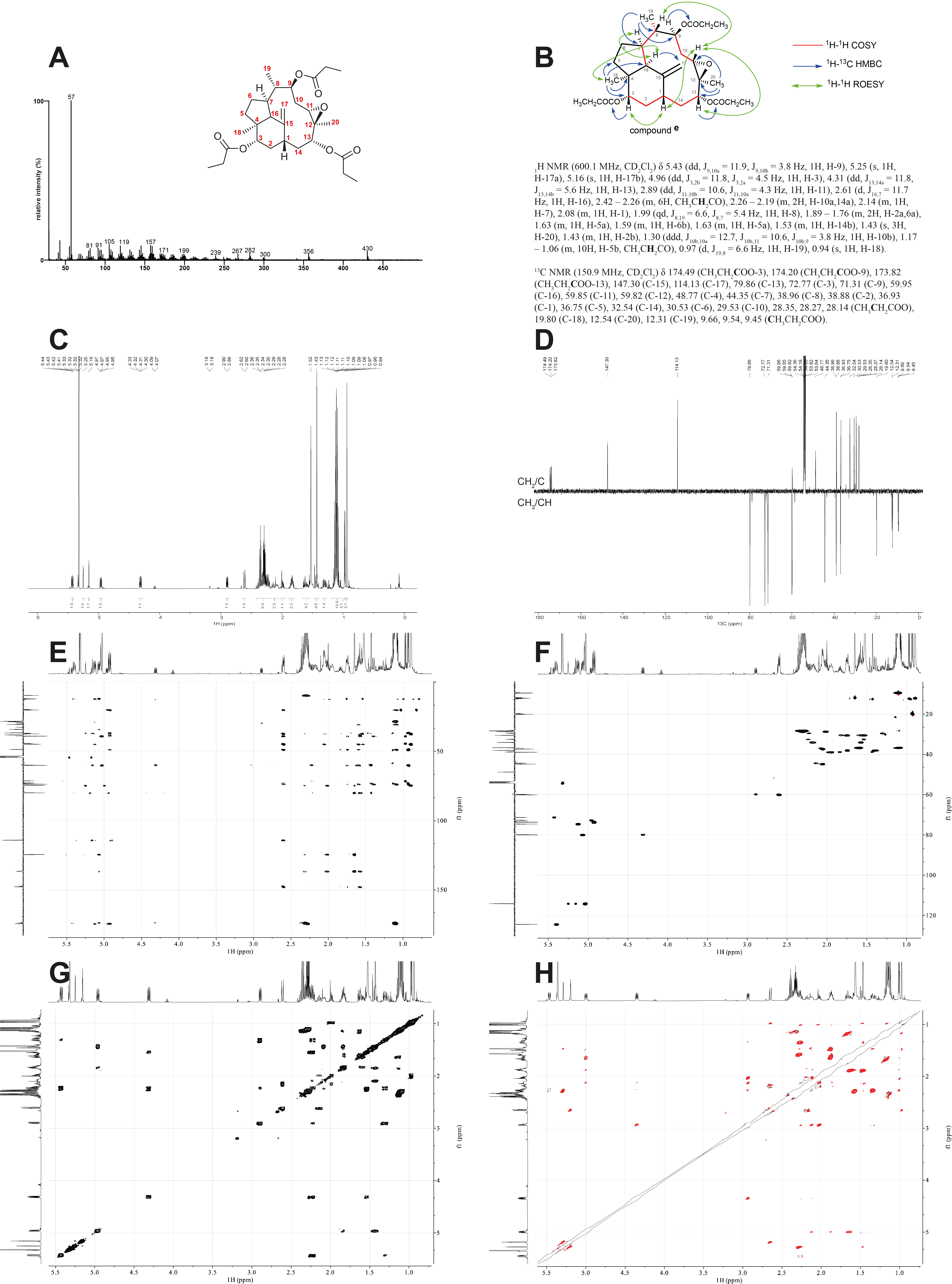

Supplement: S8 Fig — A. Mass spectrum. B. Key 2D NMR correlations and assigned 1H and 13C NMR spectra. C. 1H NMR spectra. D. APT NMR spectrum. E. HMBC NMR spectra. F. HSQC NMR spectrum. G. COSY NMR spectra. H. ROESY NMR spectrum. Complete NMR data are available from OSF (https://osf.io/rkdy9). (TIF) [file pbio.3003648.s008.tif]

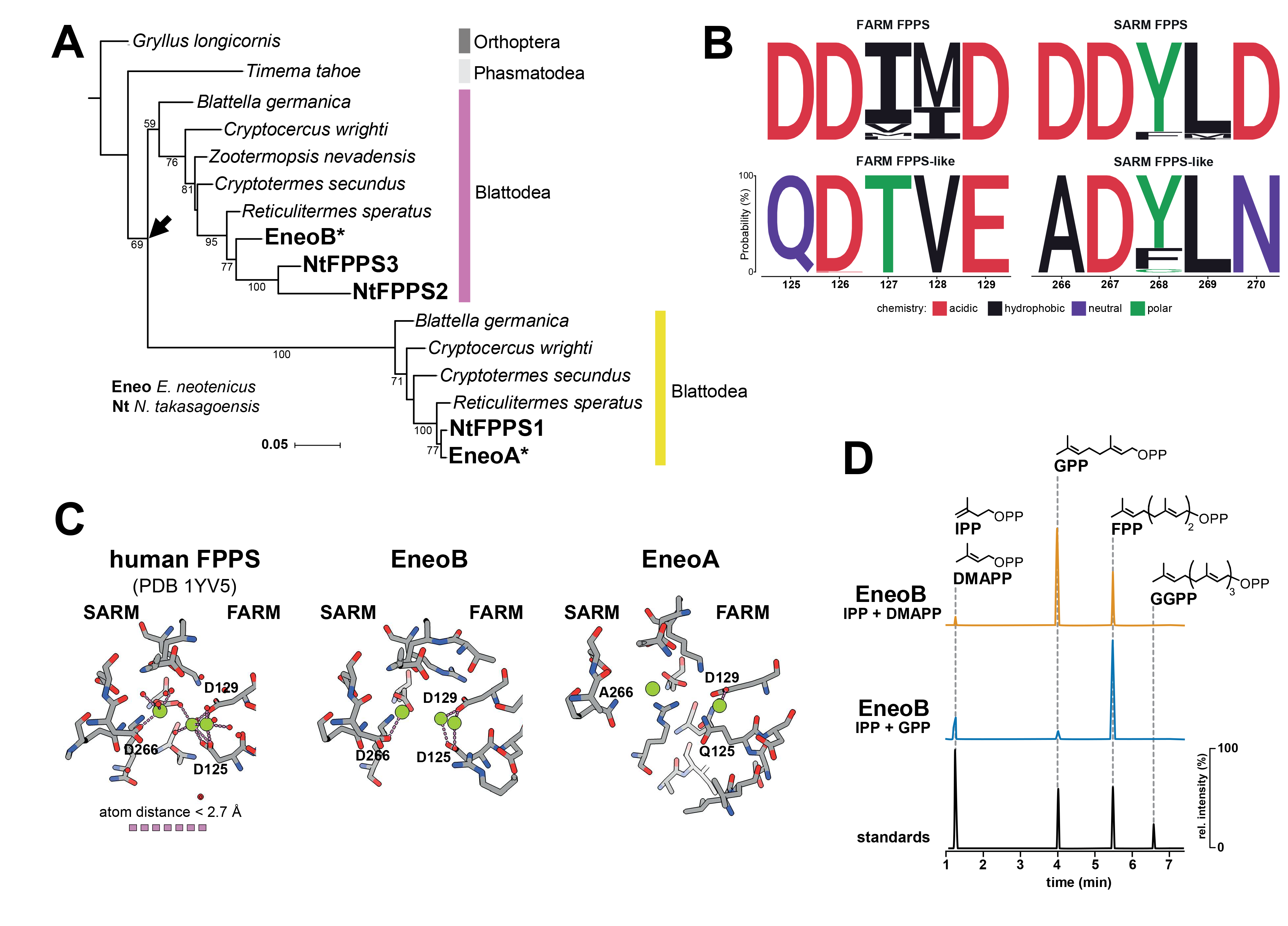

Supplement: S9 Fig — A. Phylogenetic tree inferred from amino acid sequences of FPPS homologs identified in Blattodea (five termite and two cockroach species) and two other polyneopteran orders, Orthoptera and Phasmatodea. The maximum-likelihood tree was reconstructed with IQ-TREE 2 using JTT + G4 model; bootstrap values (500 replicates) greater than 50 are shown as node support. Arrow marks the FPPS duplication predating the diversification of Blattodea. Asterisks highlight the two paralogous sequences EneoA and EneoB from Embiratermes neotenicus studied here with respect to their function. Accession numbers are provided in S2 Table. B. FARM and SARM motif sequence logos in FPPS- and FPPS-like proteins, as inferred from 197 amino acid sequences of Blattodea listed in S3 Table. C. A crystal structure of human FPPS active site and a model of the active site in EneoB and EneoA proteins showing coordinated Mg2+ ions. D. HPLC chromatogram demonstrating the IDS activity of EneoB. The purified enzyme was incubated with isopentenyl pyrophosphate (IPP) and dimethylallyl pyrophosphate (DMAPP), IPP and geranyl pyrophosphate (GPP), or IPP and farnesyl pyrophosphate (FPP). Only the substrate combinations giving rise to any prenyl pyrophosphate products are shown. The chromatograms visualize the selected m/z 245.00, 313.06, 381.12, and 449.19. No TPS activity was recorded in EneoB and no TPS or IDS activity was observed in EneoA. Complete chromatographic data and phylogenetic tree file are available from OSF (https://osf.io/rkdy9). (TIF) [file pbio.3003648.s009.tif]

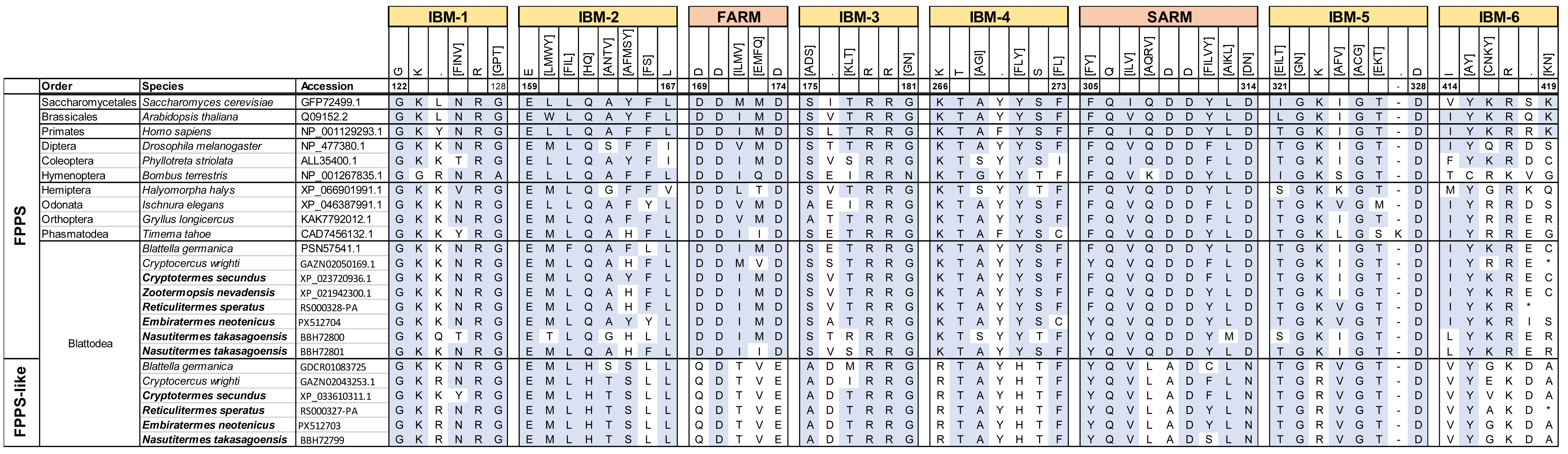

Supplement: S10 Fig — Motif boundaries and sequence regular expressions are displayed as defined by Rebholz and colleagues (2023) for insect IDS sequences. Residues in consensus with the regular expression are shaded blue. Termite species are displayed in bold text. IBM, IPP-binding motif. FARM, first aspartate-rich motif. SARM, second aspartate-rich motif. (TIF) [file pbio.3003648.s010.tif]

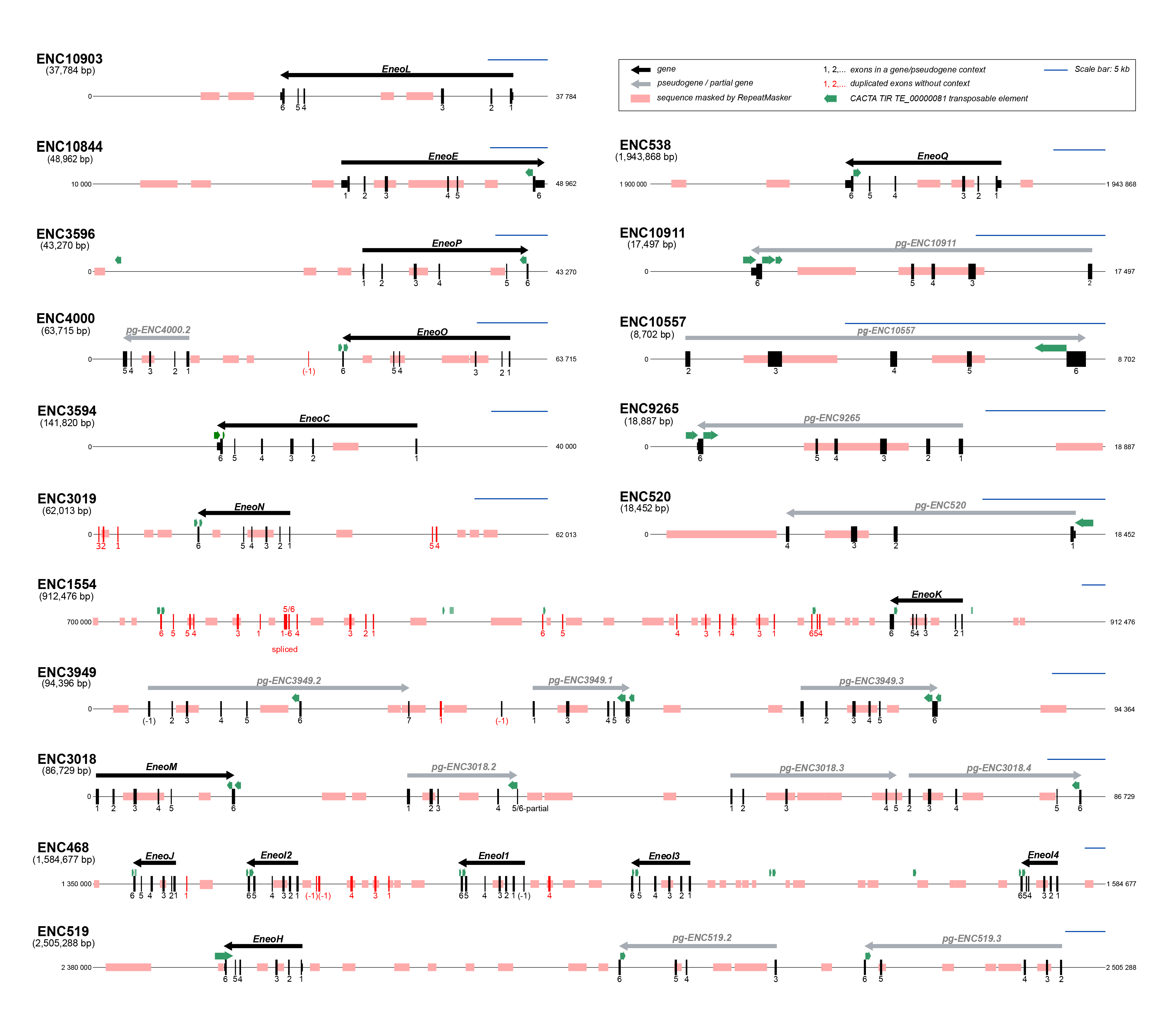

Supplement: S11 Fig — The figure shows gene organization of full or partial genomic contigs (boldfaced, starting with prefix ENC and contig size in brackets) represented by horizontal line with position coordinates at both sides. Exons are numbered and shown as black (genes), gray (pseudogenes), or red boxes (chimeric duplicates), regions corresponding to genes and pseudogenes are marked with black or gray arrows while regions masked by RepeatMasker at genome-wide scale are shown as pink boxes. Sequences homologous to CACTA TIR TE_00000081 DNA transposon predicted with the EDTA pipeline are marked with green arrows. Blue scale bar represents 5 kb. (TIF) [file pbio.3003648.s011.tif]

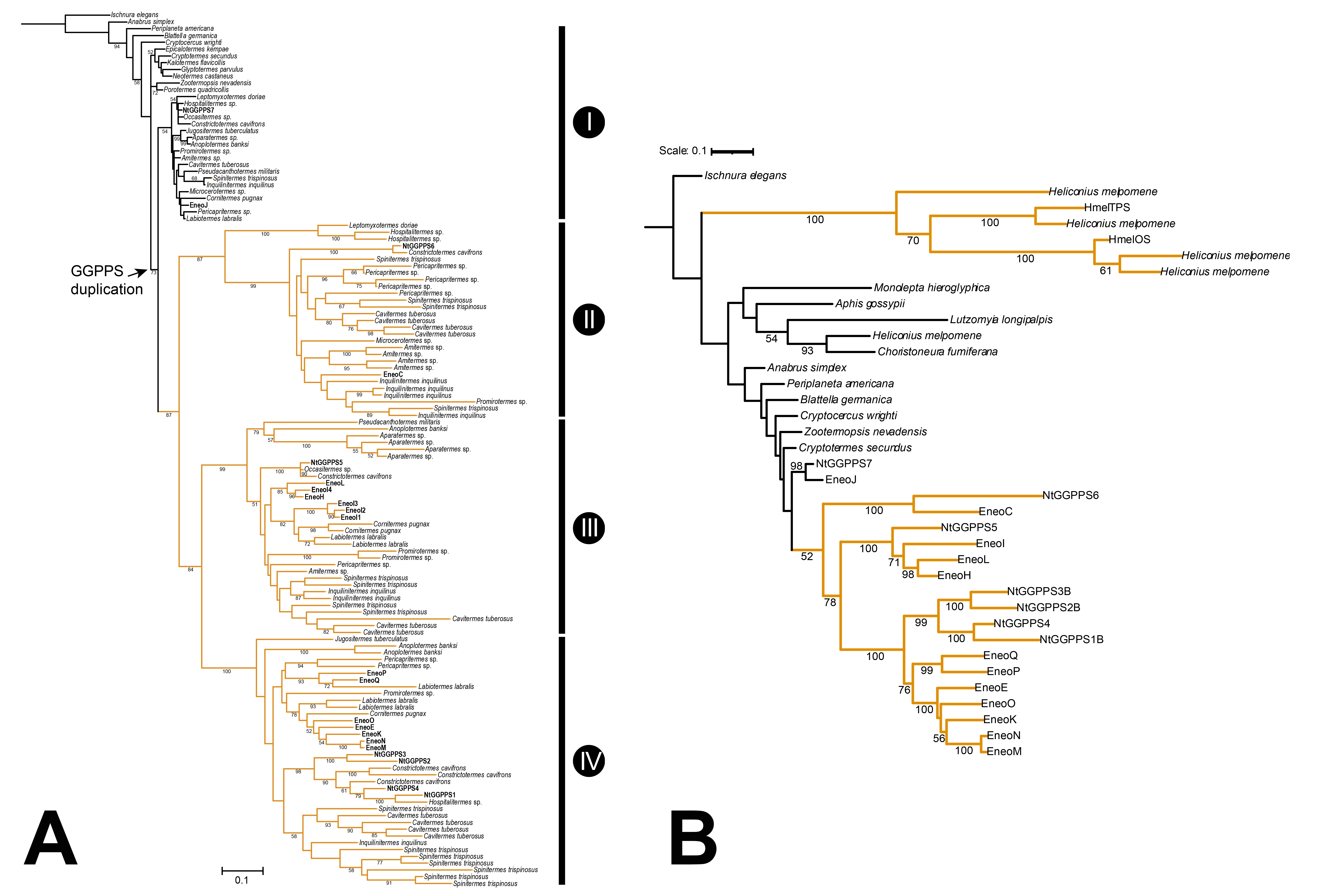

Supplement: S12 Fig — A. Taxon sampling focusing on Neoisoptera-specific GGPPS multiplication. The tree was reconstructed using IQ-TREE with the JTT + F + R4 substitution model; bootstrap values (1,000 replicates) greater than 50 are shown as node support. The tree was rooted with a dragonfly GGPPS sequence (Ischnura elegans). Roman numerals denote the paraphyletic GGPPS clade I and monophyletic GGPPS-like clades II–IV. See Supplementary Table S6 for source sequences. The tree file is available from OSF. B. Origin of GGPPS duplications in Neoisoptera and the butterfly genus Heliconius. A maximum-likelihood phylogenetic tree was inferred from an alignment of amino acid sequences using IQ-TREE with the JTT + I + G4 substitution model; bootstrap values (1,000 replicates) greater than 50 are shown as node support. The tree was rooted with a dragonfly GGPPS sequence (Ischnura elegans). See S6 Table for source sequences. The tree file is available from OSF (https://osf.io/rkdy9). (TIF) [file pbio.3003648.s012.tif]

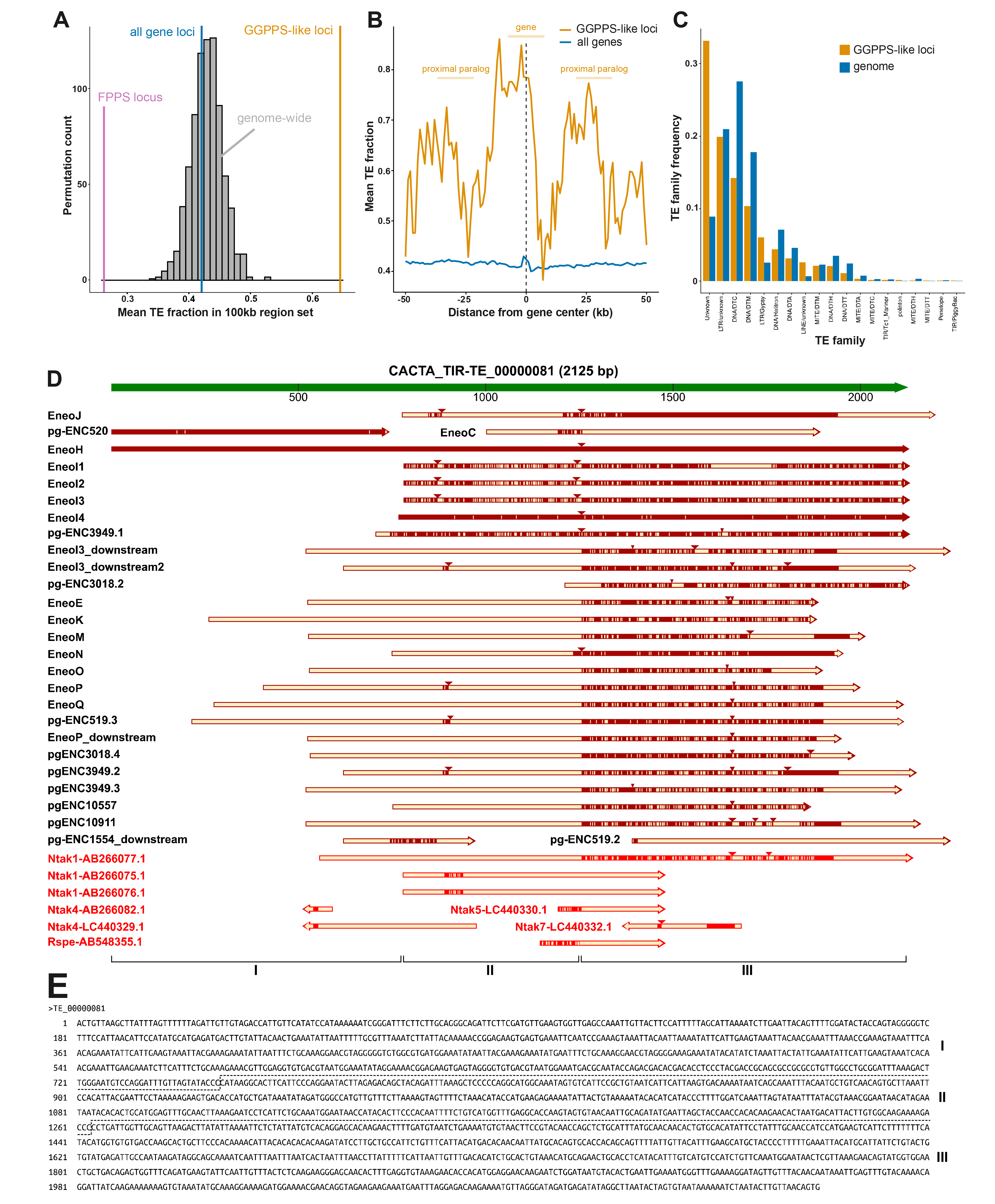

Supplement: S13 Fig — A. Mean TE frequency in 100 kb windows surrounding GGPPS-like paralogs (orange line), FPPS-like paralogs (pink line), all protein-coding genes (blue line) and genomic background represented by a histogram of TE frequencies in 100 kb genomic regions generated within 1,000 random permutations (gray bars). B. Mean TE density according to distance from the gene center (dashed vertical line) in GGPPS-like loci (orange) and protein-coding genes (blue). TE frequencies were calculated in strand-oriented 1,000 bp bins, satellite peaks in GGPPS-like loci correspond to proximal paralogs located in the same genomic locus with a mean distance of 23 kb from the gene. C. TE family representation in 100kb windows surrounding the GGPPS-like loci (orange) compared to the abundance on whole-genome level (blue). Graph bars represent the relative frequencies of TE families among all predicted TEs. D. Sequence conservation of the CACTA TIR DNA transposable element linked with GGPS-like genes. Sequences corresponding to CACTA TIR TE_00000081 predicted with RepeatModeler (green) and homologous sequences found in the proximity of GGPPS-like genes and pseudogenes in E. neotenicus (yellow) are shown as arrows, conserved regions are represented by dark color. Red triangles above sequence diagrams represent indels relative to the CACTA TIR TE_00000081 reference. Orthologs from related species N. takasagoensis (Ntak) and R. speratus (Rspe) found by BLAST search against the complete NCBI nucleotide collection restricted to Blattodea are included. E. The sequence of CACTA TIR TE_00000081 predicted with RepeatModeler, latin numbers I–III are used to specify three regions with various levels of conservation. Custom code used for A–C is available from OSF (https://osf.io/rkdy9). (TIF) [file pbio.3003648.s013.tif]

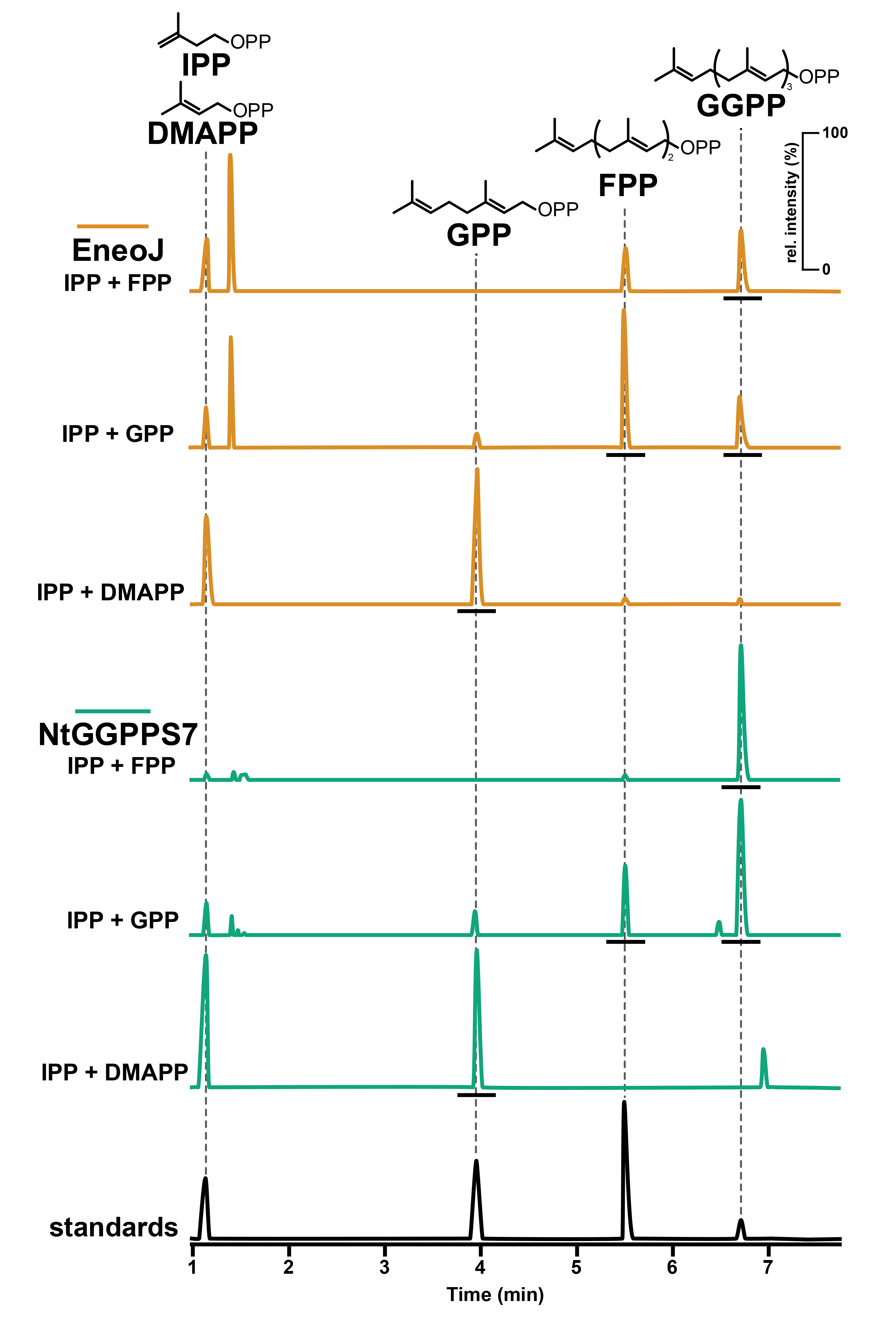

Supplement: S14 Fig — The purified enzymes were incubated with IPP and DMAPP, IPP and GPP, or IPP and FPP. The chromatograms visualize the selected m/z 245.00, 313.06, 381.12, and 449.19. The products resulting from IDS activity are underlined with black lines. Complete chromatographic data are available from OSF (https://osf.io/rkdy9). (TIF) [file pbio.3003648.s014.tif]

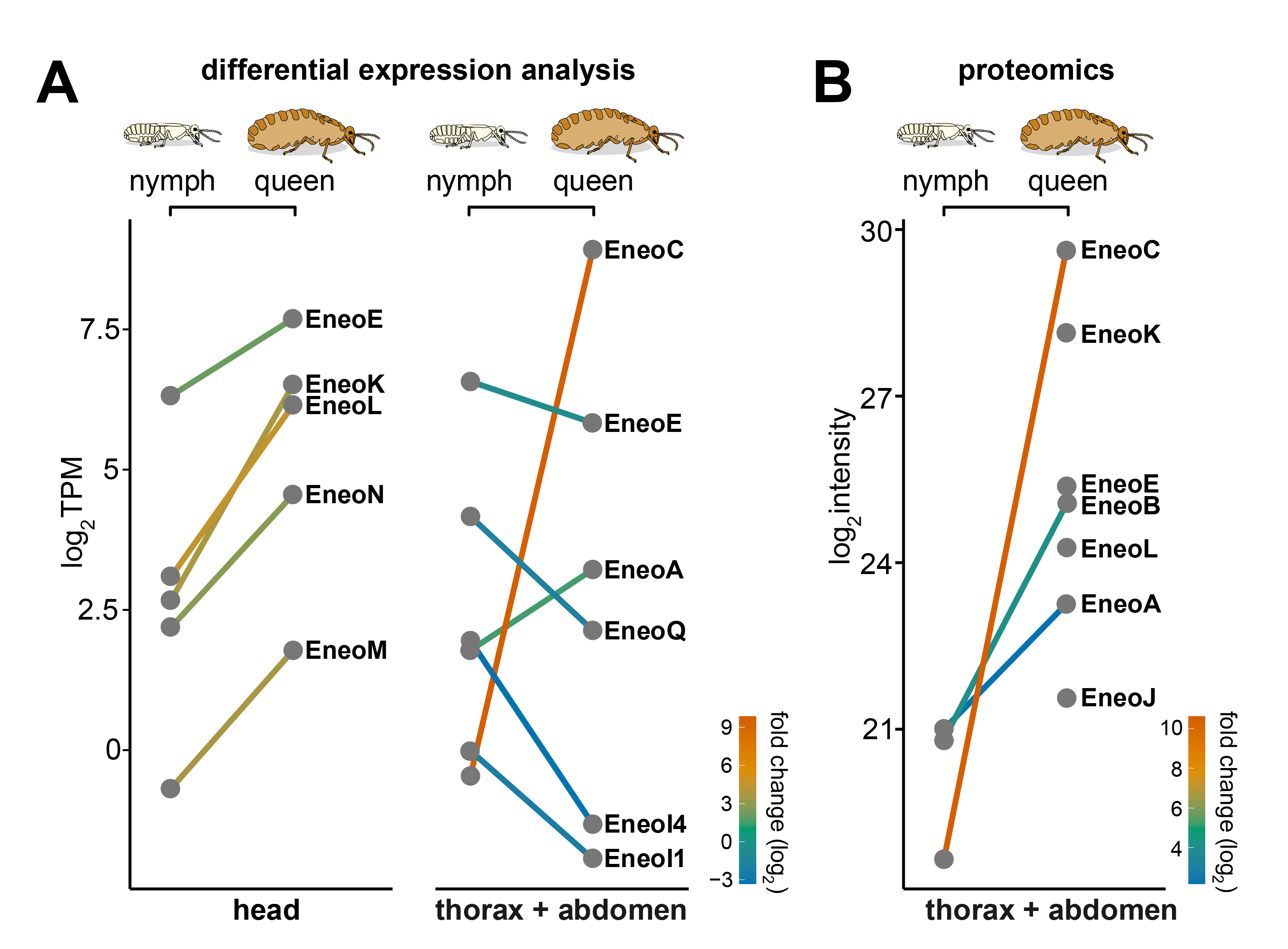

Supplement: S15 Fig — A. Differential expression analysis comparing transcript abundances of IDS and IDS-like genes in heads or thoraces and abdomens of neotenic queens and queen-destined 4th stage female nymphs. Mean log2 TPM values represent three biological replicates, colors of connecting lines correspond to log2 fold changes between the two castes. Only genes which showed significant regulation between the two castes in DESEq analysis are presented (padj < 0.05). Expression data for all IDS/IDS-like genes are listed in S8 Table. B. Quantitative proteomic analysis of IDS/IDS-like proteins in thoraces and abdomens of queen-destined 4th stage female nymphs and neotenic queens. Mean log2 intensity values represent four biological replicates. Colors correspond to log2 fold changes between the two castes. Only proteins which conform to the permutation-based FDR 0.05 are shown; points with no connecting lines represent proteins which were only detected in samples from queen thoraces and abdomens. Results of proteomic analysis for all IDS/IDS-like proteins from all tissues are listed in S9 Table. (TIF) [file pbio.3003648.s015.tif]

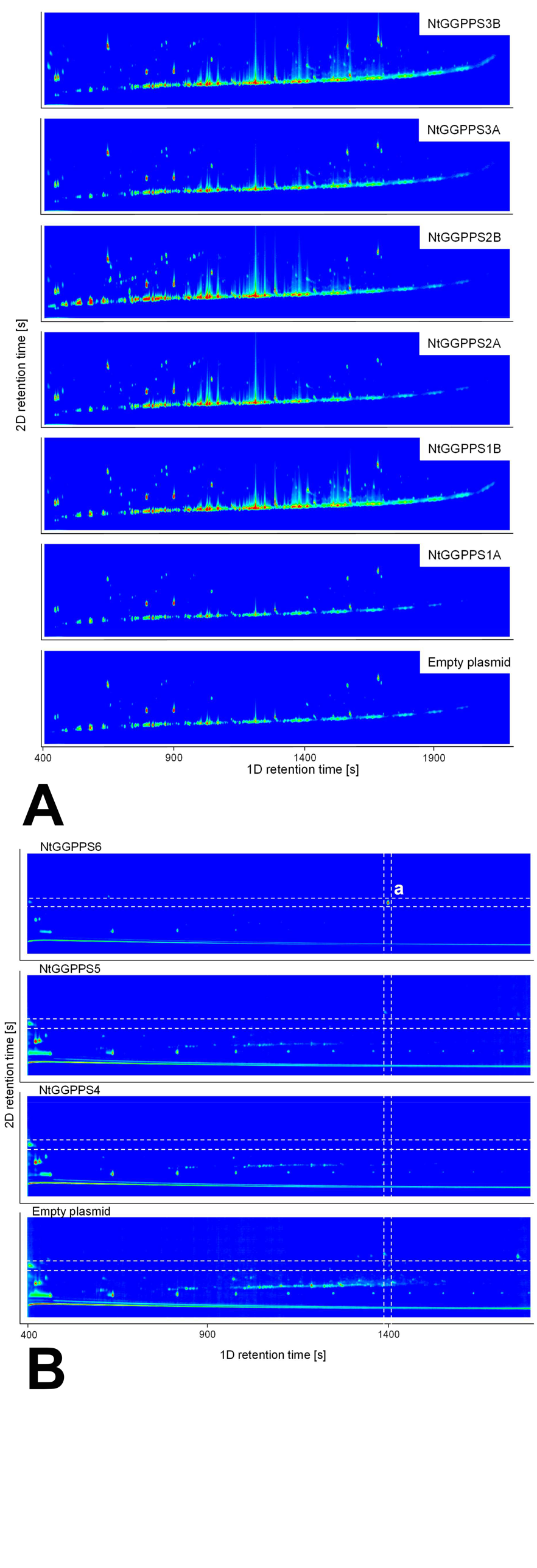

Supplement: S16 Fig — Sequences were expressed in S. cerevisiae strain JWY501. 1 mL of culture was extracted with 1 mL of hexane and 1 μL aliquot of hexane extract was measured by comprehensive gas chromatography mass spectrometry. No specific products were detected in any of the strains apart from neocembrene (a) in the NtGGPPS6-expressing strain. A. NtGGPPS1A- NtGGPPS3B. B. NtGGPPS4-NtGGPPS6. Complete chromatographic data are available from OSF (https://osf.io/rkdy9). (TIF) [file pbio.3003648.s016.tif]

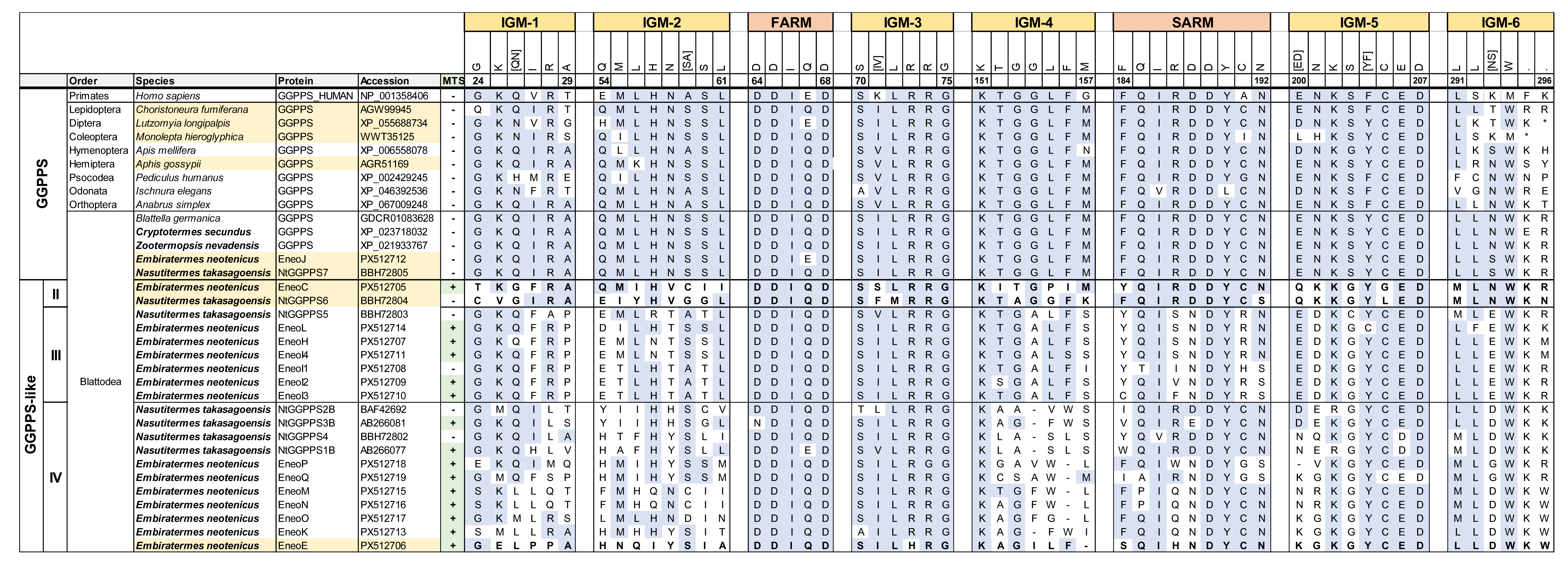

Supplement: S17 Fig — Boundaries of motifs are displayed as defined by Rebholz and colleagues [34]. Insect GGPPS-specific regular expression for each motif was generated based on the canonical insect GGPPS sequences displayed in the alignment using MEME (v5.5.7). Residues in consensus with the regular expression are shaded blue. Termite species are printed in bold text, amino acid residues for confirmed TPS proteins are set in bold. MTS, presence or absence of a mitochondrial targeting peptide as predicted by MitoFates (v1.2). IGM, insect GGPPS motif. FARM, first aspartate-rich motif. SARM, second aspartate-rich motif. (TIF) [file pbio.3003648.s017.tif]
