# Supplementary material for: Expansion of the geranylgeranyl pyrophosphate synthase gene family underlies the evolution of terpenoid biosynthesis in termites
Source: PLoS Biol. 2026 Apr 27;24(4):e3003648. doi: 10.1371/journal.pbio.3003648 (PMC13143122; doi:10.1371/journal.pbio.3003648)
Supplement: Attachment — Submitted filename: RESPONSE TO REVIEWERS_R1.docx [file pbio.3003648.s032.docx]

**This file lists the reviewers’ reports in full, and the authors’ responses are set in bold.**

**Reviewer #1:**

I really like this manuscript!

The authors address the interesting and challenging subject of terpene/terpenoid biosynthesis in termites. This study sits within the fascinating area of terpene biosynthesis in insects, which shows significant differences from terpene biosynthesis in plants and microorganisms, and is in many ways more diverse and rich.

The manuscript uses the Introduction section to provide a scholarly summary of terpene biosynthesis in insects, which sets up the current study very well. Complementary expertise is applied to the subject, including organic chemistry, biochemistry and evolutionary biology to produce a very comprehensive and rigorous piece of work. Fascinatingly, it would seem that TPSs in termites have arisen from GGPPSs, whether they are used to produce sesquiterpenoids (i.e. nerolidol) or diterpenes (cembrene). This contrasts nicely with FPPS-derived diterpene synthases from other insects.

Although I have ticked the minor corrections box, I have no corrections for the main manuscript itself, which I judge to be acceptable for publications as is (I rarely select this option!). I did notice that in the supplementary section, Fig S6 C and D are labelled the wrong way round. Also, for the APT NMR data, it would be helpful to state the pos/neg phasing of the C types (CH/CH3 vs C/CH2), as this is the opposite phasing is often employed. Additionally, on the topic of NMR, it is stated that identifications are based on numerous multidimensional NMR, but these data are not shown. For completeness, it would be good to see these spectra.

**RESPONSE:**

**As for the labelling of Figs S6C and D, it is now corrected. Thank you for spotting the mistake.**

**We have included HMBC, HSQC, COSY, and ROESY 2D NMR spectra for all three identified diterpenes and indicated the APT phasing. Furthermore, we have provided structures with highlighted correlations that allowed us to identify the three novel diterpenes.**

**Reviewer #2:**

In the presented manuscript Horacek et al. describe the identification, biochemical characterization, and possible evolutionary origin of terpene biosynthesis in Neoisoptera. The authors start by chemically characterizing different specimens from the termite species *Embiratermes neotenicus* and *Nasutitermes takasagoensis*. They find the previously identified queen pheromone nerolidol end the diterpene neocembrene in the extracts from *E neotenicus* queens and two monoterpenes as well as three oxidized diterpenoids in the soldiers of *N. takasagoensis*. Next, they investigated the genomes and transcriptomes of termites for candidate terpene synthase genes. This analysis showed a neoisoptera-specific duplication of geranylgeranyldiphosphate synthases (GGPPS), which, within neoisoptera, shows great consecutive diversification. In the genome assembly of *E neotenicus*, which was generated for this study, these duplicated GGPPS-like genes occur close to transposable
elements, which are likely responsible for the numerous duplications of this gene family. Two GGPPS-like genes, which were relatively overexpressed in queens of *E neotenicus* with respect to nymphs of that species, were functionally characterized. Indeed, these two enzymes showed enantioselective nerolidol production, and neocembrene production, respectively. The authors also characterize six GGPPS-like genes obtained from *N. takasagoensis* soldier transcriptomes, of which only one was active upon heterologous expression. However, the produced enzyme was a selective neocembrene synthase, which presents a plausible precursor for the identified soldier diterpenoids. The authors additionally conduct a selection analysis, comparing selection pressures on GGPPS-like terpene synthase genes to the ones influencing canonical GGPPSs. This analysis shows a great decrease in negative selection comparing the former to the latter, pointing to a decrease in purifying selection, allowing
higher mutation rates and thus functional evolution. Finally, the authors identify a functional switch in the characterized *N. takasagoensis* terpene synthase, which abolishes the selective neocembrene production activity and results in the production of geranyllinalool, a linear product.
This study is very interesting and of importance in the field of specialized metabolism. It is very comprehensive and goes all the way from chemical and genomic analysis to characterizing terpene synthase enzymes in vitro, shedding light on their evolutionary history within termites and the genetic mechanisms on diversification. The article is clearly written and mostly easy to follow despite the described work being highly multidisciplinary. The overwhelming majority of the conclusions made in the manuscript are robustly backed by data, which is comprehensively presented in the supplementary information.
Thus, in conclusion, I recommend publication of this article after addressing some minor concerns and suggestions, which are listed below.

General comments:
Usually the identifiers for partial figures (e.g., A, B, etc) are at the top left corner of each element. Throughout this manuscript they are at the bottom left which, at least for me, is very confusing to look at. Please consider moving the identifiers to the top-left corners.
**RESPONSE: We agree and we moved the identifiers to upper left corner in all figures in the main text and in all supplementary figures.**

Details:
-Abstract: "Termites produce the most diverse array of terpenoids among metazoans, comprising over 200 structures." - This is an incorrect statement. There are thousands of structurally diverse terpenoids from sponges and corals.

**RESPONSE: Thank you for pointing out this obvious mistake. Our statement should consider only terrestrial animals. We modified the sentence accordingly.**

-Abstract: "and the precursor of polycyclic defensive diterpenes (E,E,E)-neocembrene in *Nasutitermes takasagoensis*" this has not been proven yet, please soften the language to something like "plausible precursor".
**RESPONSE: We agree and we change to “presumed precursor”.**

-Page 4 lines 63-66: In these two sentences the authors state that utilizing classical TPS genes originating from horizontal gene transfer is an alternative to utilizing classical TPS genes. I think this statement should be removed, as receiving a gene via horizontal gene transfer at an unclear point in time seems like an arbitrary distinction versus having carried in a genome for a longer (unspecified) time. Many genes have been transferred via HGT at some point in time, possibly too long ago to detect it conclusively.
**RESPONSE: We agree that the reasoning was incorrect. We now reworded the respective two sentences as follows: Like other animals, insects generally lack the “classical” TPS genes known from plants, bacteria, and fungi [13], except for a rare case of their gain via horizontal gene transfer [14]. Therefore, insects rely on alternative mechanisms to obtain terpenoids, such as sequestering them from the diet, acquiring them from microbial symbionts, or recruiting a completely unrelated enzyme family for TPS functions [15].**

-Page 4, lines 83-86: Here the authors state that neocembrene is the precursor to polycyclic, more complicated diterpenoids. To my knowledge, there is no direct evidence for this (e.g., feeding studies with labeled neocembrene showing incorporation into the diterpenoids, or enzyme characterization by in vitro assays or knockouts). So, this should be phrased more as a hypothesis. If there is indeed direct evidence, please cite this here specifically.

**RESPONSE: We agree and we changed to “hypothesized precursor”.**

-Page 7 lines 147-149: The authors conclude from the phylogenetic analysis and the biochemical inactivity that the tandem duplicated FPPS genes are not relevant in terpene biosynthesis. I don't think this can be definitively stated here. An ancestral tandem duplication preserved in all of Blattodea could point to some function, otherwise this gene might have been lost at least in some species. Apparent biochemical inactivity might result from a variety of reasons (improper protein folding, missing PTMs, incorrect substrates tested) and even if the gene from the tested species has lost its function this does not automatically mean that this find is true for all termites. Please soften the language here.
**RESPONSE:**

**The duplication of FPPS in all Blattodea and especially its retention are intriguing, and we share the view that the FPPS-like proteins might have a specific function. Our reasoning for not considering these proteins as candidate terpene synthases is based on two points. Firstly, of all Blattodea, terpenoid biosynthesis has only been documented in a subset of termites (Neoisoptera) and in the cockroach genus *Periplaneta*. We show that in Neoisoptera, terpenoid biosynthesis is linked to the occurrence of a Neoisoptera-specific GGPPS-like gene family. Similarly, in *Periplaneta*, we noted that in addition to the pan-Blattodea FPPS-like gene duplication, a distinct, *Periplaneta*-specific FPPS-like branch exists. We propose that the *Periplaneta*-specific, rather than the pan-Blattodean, FPPS-like proteins represent primary candidate terpene synthases. Secondly, as we probably did not stress enough in the manuscript, the FARM and SARM motifs in the pan-Blattodea FPPS-like proteins present substantial amino acid changes compared to the ancestral state. As a result, coordination of M(II) ions in the active site is not possible in the same way as in typical IDS and TPS proteins, compromising the ability of FPPS-like proteins to cleave the C-O bond between the allylic group and the diphosphate. To support this observation, we now include two new panels in Supplementary Figure S9. Figure S9B now shows the FARM and SARM sequence logo in Blattodean FPPS and FPPS-like proteins, and Figure S9C compares a detailed view of the active site in the crystal structure of human FPPS with bound Mg2+ ions to an analogous site modelled for *E. neotenicus* FPPS and FPPS-like proteins for which Mg2+ coordination was calculated in Chimera. In the FPPS-like protein EneoA, no Mg2+ coordination is predicted in the putative active site of the protein. The main text was modified accordingly and the Methods section lists both the generation of sequence logo from 197 FPPS and FPPS-like amino acid sequences of Blattodea and the prediction of Mg2+ binding in *E.neotenicus* proteins. To soften our claims, we reworded the closing sentence of the paragraph to “are unlikely to be involved in terpenoid biosynthesis”.**

-Figure 4: The colors for negative control and one of the chiral standards are too similar, it took me a while to make sense of the chromatograms. Consider a different, lighter shade of gray for the S standard. Both R and S could be the same color, as they are also specifically labeled. Please explicitly state in the figure caption that the absolute configuration for neocembrene was not determined in this study. As it is now the figure alone will suggest it is R in both cases as it is drawn that way.

**RESPONSE: We understand and we a) change the color of the S standard to lighter gray and b) remove the absolute configuration from all neocembrene structures both in the main text figures and supplementary figures.**

-Figure 6: Please also label T152I and WT traces for the assays of NtGGPPS6 and GGPP.
**RESPONSE: Corrected.**

-Page 16 lines 419-422: The authors describe mitochondrial targeting sequences and refer to figure F13. However, I cannot identify any reference to mitochondrial targeting sequences in this figure. Out of curiosity: Do the authors think that the GGPPS-like enzymes are indeed transported to the mitochondrion?
**RESPONSE: Thank you for pointing out the wrong reference to a Supplementary Figure which we corrected: Supplementary Figure S17 lists the presence or absence of predicted mitochondrial targeting sequences in selected termite GGPPS and GGPPS-like sequences. We have also added a note on not focusing on subcellular localization of GGPPS/GGPPS-like proteins in this study. However, we do elaborate on this intriguing topic in a manuscript which is currently in preparation and which focuses on the biosynthetic origin of the queen pheromone in *E. neotenicus*.**

-Page18 lines 463-469: While the functional switch by the single amino acid variant T152I is interesting, the presented data does not allow for a detailed analysis of the specific interactions of the amino acid sidechains in threonine or isoleucine. A very likely scenario that is not discussed here is that the initial substrate binding position is impacted in a way that either promotes attack by water, leading to linear terpene alcohols or by the double bond at C-14, leading to cyclization. Different binding modes can also explain the shift from almost racemic to strongly enantioenriched water attack in the production of nerolidol. On the other hand, it is quite unlikely that the saturated carbon side chain of isoleucine contributes significantly to the electronic stabilization of a carbocationic intermediate. Hence, I would encourage the authors to also briefly discuss steric effects on the observed enzymatic activities.

**RESPONSE: We agree that our data allow for multiple interpretations and that the lack of a crystal structure and/or a more comprehensive mutational analysis limits the strength of our hypothesis. We thank the reviewer for suggesting an alternative mechanism, which we have now included in the discussion.**
**We further agree with the reviewer that it is highly unlikely that the aliphatic side chain of isoleucine stabilizes any carbocation. This was likely due to poor phrasing on our part, and we have revised the sentence accordingly.**

Page 27 lines 699-700: What software was used for substrate docking?

**RESPONSE: We created the models with all the ligands including three magnesium cations and substrates using Alphafold 3. We did not use any other docking systems. We rephrased this section of methods to clarify this.**

Supplementary Information:
Please provide more information on the structure elucidation of the diterpenoids of *N. takasagoensis.* Currently, there is only 1D NMR data presented and a list of signals mapped to the structures. Please show the 2D-NMR spectra and specify the correlations that allowed the unambiguous structure elucidation (a figure with the typical single- and double head arrows should suffice).

**RESPONSE:**
**We have included HMBC, HSQC, COSY, and ROESY 2D NMR spectra for all three identified diterpenes. Furthermore, we have provided structures with highlighted correlations that allowed us to identify these compounds.**

**Reviewer #3:**

This manuscript provides an interesting and detailed description of terpenoid biosynthesis and its evolutionary origins in termites. The text was well-written and the figures (except for some minor issues described below) were effective for the information presented. The data presented appear to provide good support for the conclusions drawn.

Main Text:
Line 73: Reference 20 describes TPS activity from a GPPS, not an FPPS as indicated in the text. See also: 10.1073/pnas.0503277102.

**RESPONSE:**

**The improper wording resulted from *I. pini* GPPS/TPS consistently clustering with FPPS sequences in IDS/TPS phylogenies. We have changed the sentence to „(…) TPSs are paralogous either to geranyl and farnesyl pyrophosphate synthases (GPPS and FPPS [18–23]) or geranylgeranyl pyrophosphate synthases (GGPPS [24,25]).“**

Line 221: This is the first mention of EDTA as a software. Perhaps spell out: Extensive de novo TE Annotator (EDTA).

**RESPONSE:**

**The abbreviation now first occurs a few lines higher in the text and is spelled out.**

Line 241: Which other substrates?

**RESPONSE:**

**A list of substrates not accepted by EneoC was appended in parentheses: „(GPP or GGPP in TPS assays, IPP combined with DMAPP, FPP, or GGPP in IDS assays).“**

Line 292: Incorporate HyPhy RELAX into the previous sentence, i.e., "...with HyPhy RELAX. Our analysis showed..."

**RESPONSE:**

**This was re-written as suggested.**

Line 550: alpha symbol missing

**RESPONSE: Corrected, thank you.**

Line 877-879: The annotated genome would be utilized by a much wider audience if it was submitted to a public data repository such as NCBI rather than "hidden" in the ASEP data repository that only readers of your published manuscript are likely to discover. I.e., As is, this data will not become incorporated into NCBI's BLAST databases. I strongly encourage the authors to submit the annotated genome to NCBI.
**RESPONSE:**

**We fully agree with the reviewer that it would be much more beneficial to submit the genome assembly to a common public repository such as NCBI and in fact, we are definitely planning on doing so. There are two main reasons why we decided to use the ASEP repository at this stage: i) Because of national and institutional data management policy and open data philosophy which encourage us to deposit all available resources in national repositories, and ii) the main reason, we plan to improve the draft genome with additional DNA sequencing followed by multiple polishing stages prior to submitting it to NCBI public repository. The quality of current assembly is high enough to enable identification of highly duplicated GGPPS-like paralogs which is the goal of the present study, but we feel that there are many improvements that should be made to reach high-quality publicly available genomic reference. Some of these issues are currently being solved, but it will take some more time to get an assembly version suited for NCBI submission.**

Supporting Figures issues:
Fig. S6A, S7A, and S8A have issues will displaying m/z labels, and the lines themselves.

**RESPONSE: This must be an issue in displaying the Supplementary File made in MS Word. In the submitted file, the lines and m/z labels look OK. Therefore, we add to this revision a pdf file of the supplement.**

Fig. S6C and D appear to be reversed relative to their legend information.

**RESPONSE: Thank you for spotting this error! It is now corrected.**

Fig. S15 the termites are cut off at the top.
**RESPONSE: This must be an issue in displaying the Supplementary File made in MS Word. In the submitted file, the drawings look OK. Therefore, we add to this revision a pdf file of the supplement.**

Supporting Tables issues:
Table S1, the BUSCO scores don't indicate which parameter they are referring to.

**RESPONSE: Detailed results from BUSCO analysis were added to Supplementary table S1 according to the reviewer’s suggestion**.

Table S6, is labeled Table S5 at the top of the worksheet.
**RESPONSE: Corrected, thank you for spotting the mistake.**

**Reviewer #4:**

The manuscript titled "Evolutionary origin of terpenoid biosynthesis in termites" examines the genetic and molecular basis of terpenoid biosynthesis in two termite species. Termites are known to produce more than 200 types of terpenoids; however, the biosynthetic pathways and the enzymes involved in their production have not been well elucidated.
The authors focused on two termite species, *Embiratermes neotenicus* and *Nasutitermes takasagoensis*, and analyzed the terpenoid compounds produced in different castes of each species. They combined genome sequencing with tissue- and caste-specific transcriptome analyses to identify candidate genes involved in terpene biosynthesis. In subsequent phylogenetic analyses and functional assays of selected candidate genes, the authors found that some members of a clade of GGPPS-like genes possess TPS activity and are capable of synthesizing terpenes detected in the studied termite species.
Furthermore, the authors report that the GGPPS-like clade experienced extensive gene duplication and that these genes tend to harbor higher densities of TEs in their genomic regions. Additional evolutionary analyses suggest that genes in the GGPPS-like clade tend to experience more relaxed purifying selection and that the branch leading to this clade shows evidence of positive selection. Based on these findings, the authors conclude that gene duplications, potentially associated with TE enrichment, followed by subsequent diversification enabled termites to produce a diverse array of terpenes.
Overall, the manuscript is well written and I enjoyed reading it. Knowledge of terpene biosynthesis in insects has been accumulating in recent years, and this study provides an important contribution toward understanding the genetic and molecular basis of terpene biosynthesis in termites. While I am convinced that the identified genes indeed possess TPS activity in termites, I have several major concerns regarding the evolutionary interpretation of the results, as outlined below.

Major comments

1. The contribution of GGPPS-like gene duplication to terpene diversification remains unclear.
One of the central claims of this manuscript is that the duplication of the GGPPS-like clade, followed by diversifying selection, led to the diversification of terpene synthases in termites. The authors indeed demonstrate that several members of the GGPPS-like gene family exhibit TPS activity, and evolutionary analyses detect diversifying selection acting on this clade.
However, the genes that were functionally characterized in this study represent only a subset of the entire GGPPS-like clade, even within the two focal species examined here. Moreover, among the tested genes, a substantial proportion did not exhibit TPS activity. Therefore, while the current results clearly demonstrate that some GGPPS-like genes have acquired TPS activity, they do not yet fully support the broader conclusion that the expansion of the GGPPS-like clade itself underlies the remarkable diversity of terpene biosynthesis in termites. Additional functional characterization of GGPPS-like paralogs or a more cautious interpretation of the evolutionary implications would strengthen the manuscript.
**RESPONSE: We agree that the lack of tangible TPS activity in the remaining *N. takasagoensis* GGPPS-like proteins is in part intriguing. Even though we attempted the functional characterization multiple times and in diverse experimental conditions, we still cannot rule our technical reasons for the lack of activity, such as improper folding or non-native oligomeric organization of the proteins. In *E. neotenicus*, the functionally characterized TPSs cover the diversity of the major terpenoids produced by queens (nerolidol + neocembrene), the other terpenes only being detected as traces. Most other GGPPS-like genes could be detected both in expression and proteomic data, and their structure suggests the presence of key IDS/TPS motifs, suggesting that they may be involved in terpenoid biosynthesis as well. The situation is slightly more frustrating in *N. takasagoensis*, since among the GGPPS-like paralogs, we did not identify any producing monoterpenes, though all of the 6 GGPPS-like proteins are specifically enriched in the terpene-producing frontal gland of soldiers. By contrast, the identification of neocembrene synthase NtGGPPS6 from soldiers indeed is important for explanation of termite terpenoid diversity, since neocembrene is the putative precursor of defensive polycyclic diterpenes which make up roughly one half of the termite terpenoids. As we state in the discussion section, an involvement of other enzyme families is likely in the last biosynthetic steps.**

**We dedicated several years to attempts at uncovering the function of the remaining GGPPS-like candidates in *N. takasagoensis* and we feel we do not see many further sensible options which would change the workflow and lead to potential TPS activity detection. Therefore, as suggested by the reviewer and upon advice of the editors, we try to soften the language and be careful in drawing the evolutionary conclusions throughout the text.**

2. The evidence supporting the involvement of TEs in GGPPS-like gene duplication is insufficient.
The authors suggest that the expansion of the GGPPS-like clade may have been mediated by TEs because TE sequences are frequently found near GGPPS-like loci. However, the analyses presented do not clearly demonstrate that TE density around these loci is enriched relative to the genome-wide background distribution. Although the manuscript mentions a comparison with FPPS loci, the supporting data are not shown, making it difficult to evaluate whether the observed pattern is statistically meaningful.
**RESPONSE:**

**We thank the reviewer for this insightful feedback, which helps to strengthen the manuscript. In reaction to the comment, we performed a set of additional TE enrichment analyses. We compared mean TE densities in regions surrounding GGPPS-like and FPPS-like genes and compared them with those of loci surrounding protein-coding genes and genomic regions of a similar size generated through 1,000 random permutations. To further support our observations, we studied the strand-oriented TE distribution in 100 kb windows surrounding GGPPS-like genes and pseudogenes with 1,000 bp resolution and compared it to the mean distribution in all protein-coding genes. Last but not least, we studied TE family representation in GGPPS-like loci compared to the whole genome. The resulting plots were added to modified Supplementary Fig.13 as panels A, B and C. All additional analyses were described in the method section “5.4.5 Transposable elements prediction in the genome of *E. neotenicus*” and commented on in chapters “2.4 GGPPS and GGPPS-like genomic loci are rich in transposable elements (TE)” and “3. DISCUSSION”. We believe that these newly introduced arguments provide a stronger support of potential TE involvement in GGPPS-like gene duplications.**

Furthermore, the Discussion suggests that the presence of MTS in GGPPS-like proteins supports the TE-mediated duplication hypothesis. However, the observed distribution of MTS could also arise if the ancestral gene of the GGPPS-like clade acquired an MTS after a gene duplication event, regardless of whether the duplication was mediated by TE activity. Therefore, the presence of MTS alone does not appear to provide strong support for the proposed TE-mediated duplication scenario. A more direct comparison of TE density with genome-wide expectations or analyses of duplication structures would be necessary to substantiate this hypothesis.

**RESPONSE:**

**This was an unnecessary overstatement indeed. We have reworded the text, now linking the possible acquisition of an MTS to an early gene duplication event (instead of an early transposition event).**

3. The evolutionary position of GGPPS-like genes is not sufficiently discussed in the broader context of insect TPS evolution.
In this study, the evolutionary analyses of GGPPS and GGPPS-like genes are largely restricted to termite lineages. However, terpene synthases derived from IDS have been reported in multiple insect lineages. Therefore, it would be informative to place the termite GGPPS-like genes within a broader phylogenetic context that includes TPS and IDS genes from other insect taxa.
Such a comparison would clarify whether the GGPPS-like genes represent a lineage-specific innovation in termites or whether they are related to TPS genes previously identified in other insects. Including a broader phylogenetic framework would help strengthen the evolutionary interpretation and highlight the novelty of the findings.
**RESPONSE:**

**So far, the only other reported GGPPS-like TPSs in insects are those from *Heliconius melpomene* butterfly. The phylogenies shown in our manuscript illustrate the emergence of a GGPPS-like gene family in the common ancestor of Neoisoptera and indirectly support the concept of independent evolutionary trajectories leading to acquisition of terpenoid biosynthetic capacities in termites and *Heliconius* butterflies. We realize that a direct comparison including sequences from both lineages may make the independent origins more explicit. Therefore, we have now reconstructed an expanded phylogenetic tree including GGPPS and GGPPS-like sequences from *H. melpomene* as well as four sequences of functionally characterized GGPPSs from four different insect orders. The Supplementary Figure S12B clearly shows that the duplications giving rise to the GGPPS-like genes occurred independently in the two lineages.**

Minor comments
Line 607: Genome assemblies are usually polished using genomic resequencing reads rather than RNA-seq data. Polishing a genome with RNA-seq reads may be problematic because transcript reads originate from expressed genes only and can bias correction toward highly expressed transcripts. In addition, this approach may potentially collapse sequence differences among closely related gene copies or paralogs, which is particularly concerning in the case of expanded gene families such as the GGPPS-like genes analyzed in this study.
**RESPONSE:**

**We are aware of potential issues and limitations related to genome polishing with RNA-seq data, and we proceeded very carefully to mitigate any adverse effects by evaluating polished sequences with multiple doublechecks in original contigs, all with special emphasis on GGPPS-like loci. The original grant-supported project was based on gene discovery in RNA-seq data, which at one point was found insufficient due to an increasing number of paralogs. Due to limited funds, we assembled the genomic reference from Oxford Nanopore long reads alone. Given that the sequencing strategy allows efficient scaffolding but suffers from increased number of basecalling errors, we decided to improve the overall base accuracy and correct composition of homopolymeric regions using all available data. Although we were aware of the potential risk of loss of minor paralogous isoforms, we hope that the identification of as many as 28 different GGPPS-like loci has reduced these concerns. In any case, as mentioned in the response to reviewer 3, we are still working on assembly improvement including genome resequencing and additional polishing steps.**

EneoE is still expressed at a certain level in nymphs, although almost no nerolidol or neocembrene was detected in this caste (Fig. 1A and C). Could EneoE be involved in another pathway that synthesizes other terpene-related compounds?

**RESPONSE:**

**Although we did observe *EneoE* transcription in nymphs, the presence of EneoE protein in nymph abdomens and thoraces has not been detected by the proteomic analysis (Supplementary Fig. S15B, Supplementary Table S8). This is quite in line with the observed absence of neocembrene in nymph headspace and body washes. Theoretically, the involvement of EneoE in terpenoid biosynthesis in nymphs cannot be ruled out, as we also possess proteomic data from queen and nymph heads which show modest expression of EneoE in both sample types.**

What do the asterisks in Fig. 2D represent?

**RESPONSE: Asterisks mark successfully functionally characterized enzymes. This information has been added to the figure legend. Thank you for spotting this mistake.**
